# Contribution of *Starmerella bacillaris* and Oak Chips to Trebbiano d’Abruzzo Wine Volatile and Sensory Diversity

**DOI:** 10.3390/foods12051102

**Published:** 2023-03-04

**Authors:** Giorgia Perpetuini, Alessio Pio Rossetti, Noemi Battistelli, Camillo Zulli, Andrea Piva, Giuseppe Arfelli, Aldo Corsetti, Rosanna Tofalo

**Affiliations:** 1 Department of Bioscience and Technology for Food, Agriculture and Environment, University of Teramo, Via R. Balzarini 1, 64100 Teramo, Italy; 2 Cantina Orsogna 1964, 66036 Orsogna, CH, Italy

**Keywords:** *Starmerella bacillaris*, Trebbiano d’Abruzzo wine, oak chips, co-inoculum, sequential inoculation

## Abstract

In this study, six fermentation trials were carried out: co-inoculation and sequential inoculation of *Saccharomyces cerevisiae* and *Starmerella bacillaris* in the presence and absence of oak chips. Moreover, *Starm. bacillaris* strain was attached to the oak chips and co-inoculated or sequentially inoculated with *S. cerevisiae*. Wines fermented with *Starm. bacillaris* adhered to oak chips showed a higher concentration of glycerol (more than 6 g/L) than the others (about 5 g/L). These wines also showed a higher content of polyphenols (more than 300 g/L) than the others (about 200 g/L). The addition of oak chips induced an increase of yellow color (b* value of about 3). Oak-treated wines were characterized by a higher concentration of higher alcohols, esters and terpenes. Aldehydes, phenols and lactones were detected only in these wines, independently from the inoculation strategy. Significant differences (*p* < 0.05) were also observed in the sensory profiles. The fruity, toasty, astringency, and vanilla sensations were perceived as more intense in wines treated with oak chips. The white flower descriptor showed a higher score in wines fermented without chips. Oak surface-adhered *Starm. bacillaris* cells could be a good strategy to improve the volatile and sensory profile of Trebbiano d’Abruzzo wines.

## 1. Introduction

Wine is an integral element of the culture and economy of many countries. The wine industry is facing new challenges, including the production of wines with enhanced attractiveness for consumers [1]. The globalization and the rapid worldwide access to information has resulted in more knowledgeable and empowered consumers with a more sophisticated understanding of product value and a discriminating demand for quality [1]. Several works have recently reported the influence of different winemaking technologies on white wine characteristics, such as fermentation and aging in barrels [2,3,4,5]. This is a central topic, since the aging of white wines in oak barrels is an increasing strategy among winemakers. An alternative could be the addition of oak chips to wine in order to obtain wines with properties recalling a type of wine that has been aged in barrels [6]. Moreover, the use of mixed cultures of selected non-*Saccharomyces* and *Saccharomyces* strains can lead to the production of wines with more predictable and desirable characteristics [7]. Among the non-*Saccharomyces* yeasts, *Starmerella bacillaris* (syn. *Candida zemplinina*) displays interesting oenological characteristics [8,9]. This yeast species is generally isolated from grapes and fermenting grape musts, as well as from fruits, fruit-associated insects, and soil [10,11,12,13]. This yeast displays interesting oenological characteristics, including high glycerol production, reduced acetic acid concentration when inoculated with *S. cerevisiae*, poor ethanol yield from sugars, improvement of aroma complexity, ability to metabolize malic acid, tolerance of low temperatures, ability to grow at high sugar concentrations, and fructophilic character [10,11,12,13]. 

Trebbiano Abruzzese grapevine was first documented in the Abruzzo region in the 16th century, but it seems that such a variety had already been farmed there since Roman times. Trebbiano Abruzzese is one of the several variates of Trebbiano, which is the most cultivated white grapevine in Italy, and this year its 50th anniversary was celebrated [14]. Nowadays, with about 5000 ha, it is the most widely planted white grape variety in the Abruzzo area, producing 145,323 hL of wine and bringing in 6.20 million euros of revenue (https://www.qualigeo.eu/prodotto-qualigeo/trebbiano-dabruzzo-dop/). 

Therefore, the aim of this work was to further investigate the potential of *Starm. bacillaris* in order to improve the oenological characteristics of Trebbiano d’Abruzzo wine, starting from a grape variety classified as neutral. *Starm. bacillaris* CZ31 was adhered to oak chips and used in co-inoculum or in sequential inoculum with *S. cerevisiae*. The fermentation kinetics, the main oenological parameters, the chromatic characteristics, as well as the volatile profile of the obtained wine were studied. Finally, a sensorial analysis was performed. 

## 2. Materials and Methods

### 2.1. Origin of Samples

The strain *Starm. bacillaris* CZ31 and the strain *S. cerevisiae* RT73 were used. These yeast strains were isolated from Montepulciano d’Abruzzo organic must and have been previously characterized [8,15]. All strains belong to the Culture Collection of the Microbial Biotechnology Laboratory—Department of BioScience and Technology for Food, Agriculture, and Environment (University of Teramo, Italy). Yeasts were grown on Yeast Peptone Dextrose (YPD) medium (1 % *w*/*v* yeast extract, 2% *w*/*v* peptone, and 2% *w*/*v* glucose) and incubated at 28 °C for 48 h. Strains were stored at −80 °C in YPD broth supplemented with glycerol (Sigma-Aldrich, Milan, Italy) at a final concentration of 20% *v*/*v*.

### 2.2. Pilot Scale Fermentation

Six different fermentation trials were performed in this study (Table 1). Moreover, 2 different inoculation strategies were tested. In the co-inoculation trials (CO), *S. cerevisiae* and *Starm. bacillaris* were inoculated at the same time; while in the sequential inoculation (SQ), *S. cerevisiae* was inoculated after 24 h. Furthermore, oak chips (AlcoFermBrew, Poland) (8 g/L) were added in trial 3 (CO + oak chips) and trial 4 (SQ + oak chips). *Starm. bacillaris* (CZ31) strain was attached on oak chips as previously described [10]. Briefly, *Starm. bacillaris* CZ31 was inoculated (final concentration of 6 log CFU/mL) in tubes containing chips and 20 mL of pasteurized Trebbiano Abruzzese must (72 °C for 10 min). The samples were incubated for 15 days at 28 °C. In order to verify the number of attached cells, 3 chips were randomly selected and rinsed twice with a sterile solution (NaCl 0.85% *w*/*v*), placed in a tube containing 10 mL of saline solution, and scrubbed with a cell scraper for two minutes on each side. The number of sessile cells was determined by plate count. Plates were incubated for 48 h at 28 °C. Each analysis was carried out in triplicate. 

Vinifications were carried out in a cellar of the Cantina Orsogna1964, Orsogna, (Italy) during the 2021 harvest. Fermentations were carried out in 2 hL stainless-steel fermenters containing the must (sugars 189.6 g/L; pH 3.42; total acidity 5.4 g/L expressed as tartaric acid; volatile acidity 0.12 g/L expressed as acetic acid) and were inoculated with a final concentration of 10^6^ cells/mL of both yeast strains. All fermentations were carried out in triplicate at room temperature (25 ± 2 °C). When the fermentation ended, the yeast lees were left to settle for 7 days; then, wines were racked and stored at controlled temperature in the cellar for 14 days. Wines were then placed into glass bottles (750 mL), crown-sealed, and stored at 15–20 °C for up to 30 days until sensorial analyses were performed. 

### 2.3. Physical and Chemical Analysis 

The main physical and chemical parameters (ethanol, residual sugars, titratable acidity, volatile acidity, and glycerol) were monitored through a FOSS WineScan™ FT120 rapid scanning Fourier Transform Infrared Spectroscopy and the FOSS WineScan software (version 2.2.1) according to the manufacturer’s instructions. The calibration was performed with wine samples tested using the official OIV methods [16]. The pH was measured using an InoLab 730 pH meter (WTW, Weilheim, Germany). Color intensity was evaluated as the sum of absorbance at 420 nm, 520 nm, and 620 nm using a spectrophotometer (Jenway^TM^ 6305, Thermo Fischer Scientific, Milan, Italy). Clarity (L*), red/green color component (a*), and blue/yellow color component (b*), and its derived magnitudes, chroma (C*), and tone (H*), were determined by the colorimeter (Minolta, Chroma Meter CR-5, Konica Minolta, Tokyo, Japan) using glass cuvettes with a path length of 0.2 cm after clarification of the samples by centrifugation [17]. 

### 2.4. Determination of Volatile Organic Compounds 

Extraction of volatile organic compounds (VOCs) was performed by solid-phase microextraction (SPME). Gas-chromatography coupled with a mass spectrophotometry (GC-MS) analysis was performed with a gas chromatograph (Clarus 580; Perkin Elmer, Waltham, MA, USA) coupled with a mass spectrometer (SQ8S; Perkin Elmer) following the procedure previously described by Perpetuini et al. [10]. Briefly, VOCs were extracted in a 10 mL glass vial mixing 1 g NaCl with 5 mL of sample. A carboxen–polydimethylsiloxane-coated fiber (85 μm) was used (Sigma-Aldrich, Milan, Italy). Equilibration and adsorption steps were performed, stirring the samples for 30 min at 40 °C. The fiber was placed in the injector (T = 250 °C) for 15 min, and the following program was applied: 50 °C for 2 min; first ramp, 1 °C min to 65 °C; second ramp, 10 °C min to 150 °C (10 min hold); third ramp 10 °C min to 200 °C (1 min hold). Aroma compounds were identified by comparing the retention time of pure reference standards (Sigma-Aldrich) analyzed in the same conditions. The considered compounds were ethyl acetate, ethyl butanoate, ethyl propanoate, ethyl isobutanoate, ethyl isovalerate, ethyl hexanoate, ethyl lactate, ethyl octanoate, ethyl decanoate, isoamyl acetate, ethyl 2- phenyl acetate, 1- propanol, 1-hexanol, 2-phenylethanol, hexanoic acid, octanoic acid, decanoic acid, and dodecanoic acid. 2-Methyl-hexanol was used as the internal standard. The tentative identification of other compounds was performed, comparing mass spectra with MS fragmentation patterns with those present in the National Institute for Standards and Technology database (NIST version 2005), considering a similarity >85%. Compounds, for which pure reference standards were not available, were tentatively identified only based on mass spectra comparison. Identified volatile compounds were then quantified according to the calibration curve of standards run under the same conditions as the samples, while the concentrations of other volatile compounds were calculated using 2-methyl-hexanol as previously described [18]. Data were expressed as mg/L. All analyses were performed in triplicate.

### 2.5. Sensorial Analysis 

A quantitative descriptive analysis (QDA) of the wines was carried out by a trained sensory panel of 12 tasters (5 women and 7 men) between 25 and 60 years of age. All tasters had a winetasting degree and experience. The assessors were informed about the analysis as suggested in the ISO 8586-2012 regulation [19]. As the judges were already experts; the phase of the recognition of sensations and aromas perceived was not carried out. The judges were trained to evaluate the descriptors: persistence, fruity, white flowers, honey, herbaceous, vanilla, toasty, acidity, salinity, astringency, and mouth-feel. For their improvement, scaling training (ranking of solution according to concentration of descriptor) was used. In the end, the judges were trained to become familiar with the intensity scale used (0 to 10). In this way, trained judges acquired a common qualitative and quantitative frame of reference, allowing for the use of a common scale. Zero (0) indicated that the descriptor was not perceived, while a score of ten (10) was equivalent to the highest perception. Sensory tests were performed on samples at 12 °C and coded with a 3-digit number, the sample positions were casual, and the descriptive analysis was carried out in one session. Samples of 30 mL were poured in 150 mL glasses. The analysis was performed in a sensory room, under white lights in separate booths, according to ISO 8589:2007 regulation [20].

### 2.6. Statistical Analysis 

The ANOVA test was performed using XLStat 2014 software (Addinsoft, New York, NY, USA). ANOVA was applied to identify the significant differences among oenological parameters, VOCs, and sensory descriptors, and the Bonferroni correction was applied. Principal component analysis (PCA) and Hierarchical clustering of volatile compounds (HCA) were performed using XLStat 2014 software.

## 3. Results and Discussion

### 3.1. Oenological Parameters

The main oenological parameters are reported in Table 2. 

All obtained wines showed similar values of alcohol (about 11% *v*/*v*), residual sugars (about 0.6 g/L), pH (about 3.5), total acidity (about 6%), and volatile acidity (about 0.5%). Differences were detected thanks to the content of glycerol. In fact, its concentration was higher in the wines of the trials W5 and W6. The glycerol production was influenced by *Starm. bacillaris* lifestyle, with sessile cells showing a higher production than planktonic ones. Similar results have been already reported [10]. Considering that glycerol results in wine sweetness, the accumulation of such a compound could be an advantage for the inoculation of *Starm. bacillaris* adhered to oak chips both in co-inoculation and sequential inoculation with *S. cerevisiae*. The addition of oak chips (trials W3, W4, W5, and W6) resulted in an increase of total polyphenols. In fact, wines of trials 1 and 2 had a content of polyphenols of 126 mg/L and 183 mg/L, while the wines of trials W3, W4, W5, and W6 had a concentration ranging from 220 mg/L (trial W4) to 344 mg/L (trial W6). Such data could be related to the large amount of extractable phenolic compounds from wood. In fact, several studies showed an increase of these compounds in wines aged in contact with wood (barrels or chips) as a result of a transfer of polyphenols from wood to wine [21,22]. 

It is well known that wine yeasts can provoke a reduction of polyphenols thanks to the formation of weak interactions between polyphenols and yeast walls [23]. However, this effect seems to be reversible, since these compounds can be released again into the wine. This phenomenon depends on the strength of the interactions, and this strength would depend on several factors, such as mannoprotein conformation structure and composition [23]. Moreover, it should be noted that some yeast metabolites, such as pyruvic acid [24] and acetaldehyde [25,26], can react with different classes of polyphenols [27]. 

The highest values were detected in wines obtained with *Starm. bacillaris* adhered to oak chips, independently from the inoculum type. Likely, the presence of this yeast in contact with wood could induce some metabolic modifications [10,28]. Once adhered to oak chips, *Starm. bacillaris* could favor the extraction of polyphenols thanks to some specific enzymatic activities described in this species, such as cellulolytic and hemicellulolytic enzymes [28,29]. 

Moreover, microorganisms growing as sessile cells frequently express phenotypes that are different from the planktonic counterparts [10,28]. The adsorption on the cell wall could be less efficient in sessile cells, favoring the accumulation of polyphenols in wine.

The presence of oak chips influenced the color of wines. Clarity (L*) was similar in all wines. As expected, a* values (red/green color component) were all low (all negative values), thus reflecting the absence of red color in white wines. For b* values (blue/yellow color), the addition of oak chips induced an increase of the yellow color (Table 3). A similar tendency was also detected for C* values and H* with values ranging from 2.57 (trial 3) to 3.07 (trials 5 and 6) expressed by the CIELab coordinates and from 96.05° (trial 3) to 97.59° (trial 5), respectively. Similar changes in white wine color intensity over aging time in wood barrels were detected by Recamales et al. [30]. The changes detected in the present study could be related to the formation of new compounds resulting from the interaction between oak wood compounds and wine. Several studies have described the participation of phenolic aldehydes in a variety of reactions that may influence wine color evolution as well. For example, different condensation products have been described as resulting from the direct reaction of some individual oak wood phenolic aldehydes (e.g., vanillin and syringaldehyde) with wine flavanols, thus contributing to wine color and astringency evolution [31,32]. Finally, the extraction of phenolic wood compounds could also induce the increase in b* values detected in wines produced with oak chips, thus confirming the results obtained for color intensity.

### 3.2. Determination of Volatile Organic Compounds 

A total of 46 compounds were detected, belonging to the following chemical classes: esters (19), higher alcohols (8), organic acids (7), aldehydes (1), terpenes (4), ketones (2), thiols (2), phenols (1), and lactones (2). The identified compounds presented different odors, whose description was taken from a comprehensive database (www.thegoodscentscompany.com) (Table 4). 

The content of thiols and ketones was similar in all wines. 1-Propenethiol showed a concentration of about 0.3 mg/L. In general, the concentration of thiols mainly depends on the precursors’ availability in grapes, considered as “varietal compounds” [33]. Ketones were present in a similar amount: 3,3-Dimethyl-4-methylamino-butan-2-one had a concentration of about 0.05 mg/L, while β-damascenone of 0.0005 mg/L in all trials.

Higher alcohols ranged from 30.43 (trial W2) to 35.91 (trial W6). The main differences were detected for 1-hexanol (ethereal, fusel, oily, fruity, alcoholic, and sweet), and 1-octanol (waxy, green, rose, and mushroom), which were mainly present in the wines of trials W5 and W6. The oak-treated wines (W3, W4, W5, and W6) contained a concentration of higher alcohols ranging from 32.27 mg/L (W3) to 35.91 mg/L (W6), while the wines of trials 1 and 2 had a concentration of 30.43 mg/L and 30.74 mg/L, respectively. In the presence of wood, yeasts are known to consume a lower amount of assimilable nitrogen [34]. Generally, a negative correlation exists between the yeast-consumed nitrogen and the total concentration of higher alcohols [35,36]. Furthermore, the obtained results suggest that alcohols are not sorbed on wood. However, other authors suggested that these compounds could be retained by wood [37]. The differences might be related to the different grape cultivar and to wood characteristics, the amount of oak chips added, and particle size. Furfuryl alcohol was detected only in wines fermented in the presence of chips with concentrations ranging from 0.08 mg/L (trials W3 and W4) to 0.31 mg/L (trials W5 and W6). This compound does not originate from wood but is formed through the reduction of furfural, which is formed from pentoses, the main constituents of hemicelluloses [37. As expected, furfuryl alcohol was present only in oak-treated wines, which contained furfural. 

Volatile organic acids are formed during yeast fermentation. Anabolic and catabolic biogenetic pathways come into question for the occurrence of fatty acids in wines. Maybe, both the reaction mechanisms—that is, degradation owing to the β-oxidation in the initial phase of fermentation as well as the anabolic processes—play a role in the formation of these compounds [38]. Particular attention is paid to these compounds, since they have aroma descriptors, such as cheese or rancid. Organic acid concentration ranged from 9.41 mg/L (trial W1) to 11.18 mg/L (trial W3). Generally, the concentration of the different organic acids was lower in wines obtained in the presence of oak chips. Similar data have been reported by other authors [38,39], and their decreases might result from the sorption process. Because of its structure and chemical composition, wood may interact with wine, resulting in the sorption of wine aroma compounds. Organic acids are not easily oxidized in wine; therefore, their decrease suggests the existence of a sorption process that could occur directly in the wood or on the solid deposits formed as a consequence of the natural wine settling [39].

However, propanoic acid displayed an increasing trend in all wines fermented in the presence of oak chips and could be due to the hydrolysis of its corresponding ester [38]. The content of esters was influenced by both the inoculation strategy and the presence of chips. The wines of trials W1 and W2 showed a content of esters of 17.78 mg/L and 22.93 mg/L, respectively, while wines of trials W3, W4, W5, and W6 showed a content of esters of 26.53 mg/L, 32.37 mg/L, 38.58 mg/L, and 43.43 mg/L, respectively. The main esters detected were ethyl acetate (rosa, sweet, honey, and fruity), ethyl decanoate (sweet, waxy, and fruity), and 2-methylbutyl valerate and ethyl octanoate (fruity, waxy, apricot, and banana). Concerning the first aspect, the sequential inoculations resulted in an increased content of esters. Several studies reported that such a strategy could be better than co-inoculation, since it favors the development of non-*Saccharomyces* yeasts at the beginning of fermentation, resulting in wines with specific sensory properties [7,40,41]. The increase of esters in the presence of chips has been reported by other authors [38,42,43,44,45,46]. In fact, it is well known that, during the aging time, esterification and transesterification take place, which might lead to increased ester concentration in wine [7].

This may be caused by esters synthesis, due to their levels being below the equilibrium concentration at fermentation and by their release into wine during yeast cellular lysis [47]. Moreover, the ellagitannins released from chips play an important role in the wine oxidation process, since they quickly absorb the dissolved oxygen. It is thus possible that these compounds have preserved the esters from oxidative degradations. In particular, the presence of chips induced an increase of ethyl acetate, ethyl octanoate, methyl vanillate, ethyl vanillate, and diethyl succinate. Similar data were obtained by Sanchez-Palomo et al. [48], who observed the increase of vanillin derivates (methyl vanillate and ethyl vanillate) in oak-treated Verdejo wines. The increase of ethyl acetate in the presence of wood was reported also by Kyraleou et al. [44] and by Rapp and Mandery [45] in the presence of acacia chips, and might be due to the fact that this compound originates both from the wine oxidation process and from the wood. The increase of diethyl succinate was reported by Călugăr et al. [49] in Muscat Ottonel wine. The increase of some esters during aging (barrel or chips) was previously reported [43,50]. Since the water molecules were observed to be smaller as compared to other compounds, water passed through wood pores (barrel or chips) more easily. The increased concentration of some compounds during aging might be due to evaporation of the water [51]. 

The content of terpenes in wines mainly derives from the grape. Generally, they are present in low levels, but thanks to their low odor thresholds, they significantly contribute to wine aroma, conferring a floral and fruity scent [38]. Four terpenes were detected: linalool, citronellol, geraniol, and eugenol. Terpene concentration increased in the presence of oak chips. In fact, wines of trials W1 and W2 had a concentration of 3.28 mg/L and 3.91 mg/L, respectively; while those of trials W3, W4, W5, and W6 had a concentration of 4.3 mg/L, 4.58 mg/L, 5.12 mg/L, and 5.16 mg/L, respectively. Such an increase was due to the content of linalool and citronellol. The increase of citronellol and linalool in oak-treated Treixadura wines was also reported by Ferreras et al. [52]. In particular, the amount of linalool almost doubled in the presence of chips. Such an increase might be related to its origin. In fact, it could originate from the conversion of precursors during fermentation and from wood. The presence of linalool in oak chips was previously described [53]. The content of geraniol and eugenol dropped out in the presence of chips, probably because of absorption phenomena by chips, as previously suggested [54].

Oak-treated wines were characterized by the presence of two aldehydes: furfural and vanillin. The amount of furfural varied from 0.87 mg/L (trial W3) to 1.43 mg/L (trial W6), while the content of vanillin ranged from 5.56 mg/L (trial W3) to 6.98 mg/L (trial W6). According to several authors [46,55,56], these results are expected, since oak wood shows a high content of these compounds. Moreover, these compounds were mainly detected in wines obtained with *Starm. bacillaris* adhered to oak chips. As stated above, the enzymatic reservoir (e.g., cellulolytic and hemicellulolytic enzymes) of this yeast could favor its extraction from oak chips.

Oak-treated wines contained 4-vinylguaiacol (sweet, spicy, clove-like, and somewhat smoky), with values ranging from 0.32 mg/L (trial W4) to 0.98 mg/L (trial W5). Such a compound can originate from wood through the enzymatic or thermal decarboxylation from cinnamic acids [45]. 

Lactones are a sub-group of esters formed by internal esterification between the carbonyl and hydroxyl groups, resulting in cyclic compounds [57]. Butyrolactone (fruity, caramel, coconut, woody, creamy, and peachy notes) and trans-whiskey lactone (coconut, celery, and pastry) were detected only in wines fermented with oak chips. The content of butyrolactone ranged from 0.91 mg/L (trial W3) to 1.12 mg/L (trial W5), while the content of trans-whiskey lactone ranged from 1.66 mg/L (trial W4) to 1.98 mg/L (trial W5). Butyrolactone is derived both from amino or organic acids during fermentation and from oak chips [58]. Trans-whiskey lactone is a natural lactone present in fresh oak wood and is formed from the cyclization of 3-methyl-4-hydroxyoctanoic acid. Such a compound is present in oak wood as a glycoconjugate precursor—namely, galloylglucoside, glucoside, and a rutinoside derivative [59].

**Figure 1:**
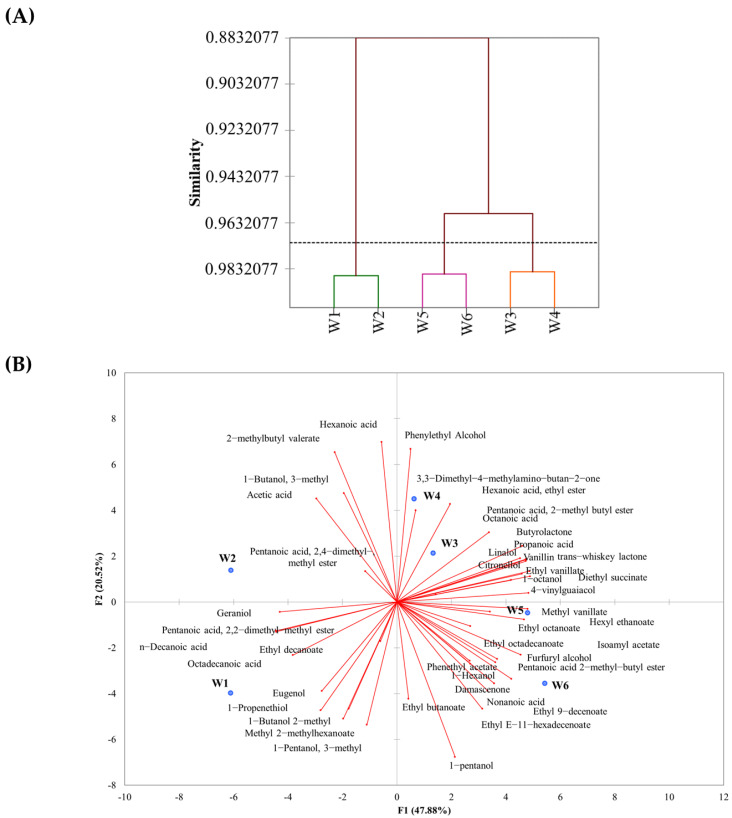
(**A**) Hierarchical clustering of volatile compounds in different wines. (**B**) PCA analysis encompassing the VOCs detected.

In order to obtain useful information from the aroma profile, PCA and HCA analyses were performed. Such analyses were performed in order to evaluate the effect of the inoculation strategy on the aroma profile of the analyzed wines and to identify the volatile compounds that best discriminate the wines. The dendrogram of HCA showed that the presence of chips as well as the different inoculation strategies (*Stram. bacillaris* aggregation state) produced wine with different aroma composition profiles (Figure 1A). PCA analysis was performed to better differentiate the wines (Figure 1B). PCA explained 68.4% of the total variance (47.88% and 20.52% for F1 and F2, respectively). Wines were differentiated into three groups. The first group contained the wines fermented without chips; the second group contained the wines fermented with oak chips and *S. cerevisiae* and *Starm. bacillaris* inoculated in the planktonic state; while the third one comprised the oak-treated wines with *Starm. bacillaris* adhered to oak chips. Wines of trials W1 and W2 were differentiated due to five compounds (hexanoic acid, 3-methyl butyl valerate, 3-methyl-1-butanol, acetic acid, and pentanoic acid 2,4 dimethyl ester).

Wines of trials W3, W4, W5, and W6 were separated on the first principal component from wines of trials W1 and W2. Wines of W1 and W2 trials were characterized by hexanoic acid, 2-methylbutyl valerate, 1-butanol, 3-methyl, acetic acid, pentanoic acid, and 2,4-dimethyl-methyl ester. Phenethyl alcohol, 3,3-dimethyl-4-methylamino-butan-2-one, hexanoic acid ethyl ester, octanoic acid, vanillin, butyrolactone, propanoic acid, linalool, ethyl vanillate, diethyl succinate, citronellol, 4-vinylguaiacol, and trans whiskey lactone separated wines of trials W3 and W4. Methyl vanillate, hexyl ethanoate, ethyl octanoate, ethyl octadecenoate, isoamyl acetate, furfuryl alcohol, phenetyl acetate, pentanoic acid, 2-methyl butyl ester, hexanol, β-damascenone, ethyl butanoate, nonanoic acid, ethyl 9-decenoate, and ethyl E-11-hexadecenoate distinguished wines of trials W5 and W6. 

**Figure 2:**
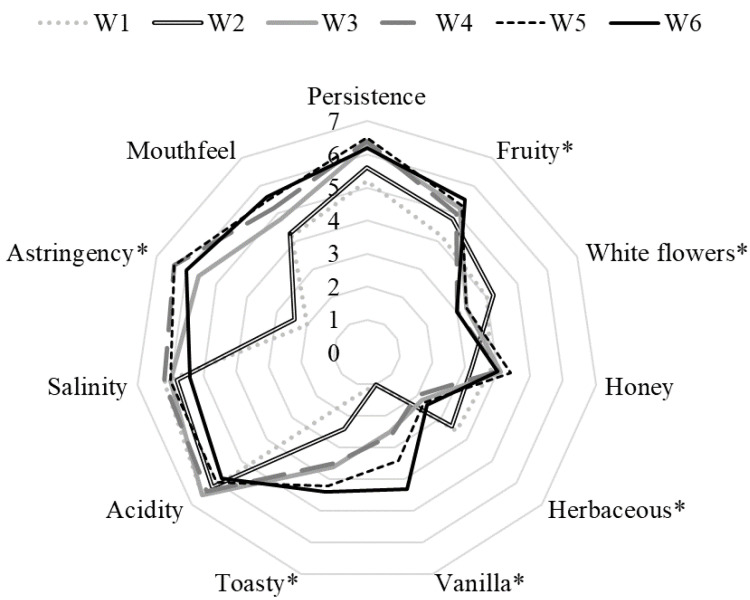
Sensory analysis of wines. * *p* < 0.05.

In order to verify if the differences detected in the sensory profile resulted in wines with different sensory traits, a sensory analysis was performed, and a radar map was generated. As shown in Figure 2, the wines of trials W1 and W2 showed the highest values for herbaceous and white flower descriptors. The toasty, honey, and vanilla sensations were perceived as more intense in wines treated with oak chips. Such findings are consistent with the presence of oak lactones and volatile phenols—i.e., the main compounds responsible for these attributes. These wines also showed a moderate astringency due to the ellagitannins released from the oak chips, and persistence due to the colloidal structure and content of polyphenols of wines. The other descriptors showed similar scores in all wines.

**Table 4:** Aroma compounds detected and their olfactory description. Data are expressed as mg/L.

Chemical Classes	Odor Description	Trials					
		W1	W2	W3	W4	W5	W6
	**Higher Alcohols**						
2-methyl-1-butanol	n.f.	2.91 ± 0.52 ^A^	1.09 ± 0.43 ^C^	1.98 ± 0.64 ^B^	0.92 ± 0.02 ^D^	0.53 ± 0.09 ^E^	1.97 ± 0.36 ^B^
Furfuryl alcohol	n.f.	n.d.	n.d.	0.08 ± 0.04 ^B^	0.08 ± 0.06 ^B^	0.30 ± 0.11 ^A^	0.31 ± 0.16 ^A^
3-methyl-1-butanol	Fusel, alcoholic, whiskey, fruity, banana	14.36 ± 3.72 ^AB^	14.97 ± 3.25 ^A^	14.29 ± 3.81 ^B^	14.85 ± 5.47 ^A^	14.57 ± 1.19 ^AB^	14.27 ± 2.56 ^B^
1-pentanol	Pungent, fermented, bready, yeasty, fusel, winey, solvent	1.12 ± 0.21 ^B^	0.44 ± 0.01 ^D^	0.67 ± 0.03 ^C^	0.43 ± 0.33 ^D^	0.98 ± 0.07 ^B^	1.54 ± 0.75 ^A^
1-hexanol	Ethereal, fusel, oily, fruity, alcoholic, sweet	0.13 ± 0.03 ^C^	0.12 ± 0.05 ^C^	0.15 ± 0.03 ^C^	0.12 ± 0.02 ^C^	3.71 ± 1.13 ^A^	2.12 ± 0.79 ^B^
3-methyl-1-pentanol	Fusel, cognac, winey, cocoa, green, fruity	0.17 ± 0.04 ^B^	0.18 ± 0.01 ^B^	n.d	n.d.	n.d.	0.27 ± 0.03 ^A^
1-octanol	Waxy, green, rose, mushroom	0.77 ± 0.13 ^C^	0.91 ± 0.01 ^C^	2.85 ± 0.01 ^B^	2.88 ± 0.01 ^B^	3.09 ± 0.01 ^B^	4.11 ± 0.04 ^A^
Phenylethyl alcohol	Sweet, floral fresh, bready, rose, honey	10.85 ± 2.16 ^D^	12.86 ± 1.72 ^B^	12.25 ± 3.19 ^C^	15.87 ± 2.34 ^A^	12.76 ± 1.81 ^BC^	11.32 ± 2.38 ^D^
**TOTAL**		**30.43**	**30.74**	**32.27**	**35.15**	**34.94**	**35.91**
	**Organic acids**						
n-Decanoic acid	Rancid, sour, fatty, citrus	3.45 ± 0.06 ^A^	3.36 ± 0.25 ^A^	2.31 ± 0.06 ^C^	2.75 ± 0.63 ^B^	2.69 ± 0.55 ^B^	2.34 ± 0.88 ^C^
Acetic acid	Sharp, pungent, sour, vinegar	0.22 ± 0.04 ^C^	0.31 ± 0.01 ^A^	0.32 ± 0.12 ^A^	0.25 ± 0.05 ^B^	0.14 ± 0.03 ^C^	0.15 ± 0.02 ^C^
Nonanoic acid	Fatty, waxy, cheesy, sweet, creamy	0.17 ± 0.02 ^C^	0.13 ± 0.01 ^CD^	0.15 ± 0.05 ^C^	0.09 ± 0.01 ^D^	0.43 ± 0.13 ^A^	0.32 ± 0.08 ^B^
Hexanoic acid	Sour, fatty, sweat, cheese	1.33 ± 0.82 ^E^	1.78 ± 0.76 ^B^	1.67 ± 0.33 ^C^	1.87 ± 0.41 ^A^	1.45 ± 0.99 ^D^	1.47 ± 0.92 ^D^
Octadecanoic acid	n.f.	1.39 ± 0.33 ^A^	1.22 ± 0.35 ^B^	0.89 ± 0.78 ^D^	0.54 ± 0.42 ^E^	0.98 ± 0.07 ^C^	0.51 ± 0.15 ^E^
Propanoic acid	Fatty, waxy, rancid, oily, vegetable, cheesy	2.62 ± 0.22 ^D^	2.75 ± 0.28 ^D^	5.08 ± 0.37 ^A^	4.18 ± 0.05 ^C^	5.13 ± 0.03 ^A^	4.48 ± 0.13 ^B^
Octanoic acid	Pungent, acidic, cheesy, vinegar	0.23 ± 0.06 ^D^	0.12 ± 0.03 ^D^	0.76 ± 0.25 ^A^	0.55 ± 0.19 ^B^	0.74 ± 0.03 ^AB^	0.34 ± 0.07 ^C^
**TOTAL**		**9.41**	**9.67**	**11.18**	**10.23**	**10.7**	**9.61**
	**Esters**						
Isoamyl acetate	Sweet, banana, fruity, ripe, estery	0.65 ± 0.16 ^C^	1.00 ± 0.45 ^B^	0.52 ± 0.23 ^C^	1.32 ± 0.18 ^B^	1.07 ± 0.75 ^B^	1.80 ± 1.33 ^A^
Phenethyl acetate	Floral, rose, sweet, honey, fruity, tropical	3.06 ± 0.99 ^D^	4.29 ± 1.62 ^B^	3.79 ± 0.13 ^C^	3.17 ± 0.45 ^C^	4.23 ± 0.21 ^B^	5.38 ± 1.06 ^A^
Pentanoic acid, 2-methyl-butyl ester	Aromatic, floral, fruity, chamomile, rose	0.52 ± 0.15 ^B^	0.64 ± 0.17 ^B^	0.61 ± 0.28 ^B^	0.41 ± 0.01 ^B^	0.98 ± 0.15 ^A^	0.73 ± 0.25 ^B^
Hexyl ethanoate	Fruity, green apple, banana, sweet	0.22 ± 0.04 ^C^	0.17 ± 0.01 ^C^	0.08 ± 0.01 ^C^	0.84 ± 0.01 ^AB^	0.65 ± 0.17 ^B^	0.94 ± 0.17 ^A^
Ethyl butanoate	Fruity, juicy, fruity, pineapple, cognac	0.32 ± 0.03 ^ABC^	0.21 ± 0.04 ^BC^	0.31 ± 0.04 ^AB^	0.21 ± 0.02 ^ABC^	0.14 ± 0.02 ^C^	0.41 ± 0.05 ^A^
Ethyl decanoate	Sweet, waxy, fruity, apple, grape, oily, brandy	3.45 ± 0.11 ^AB^	3.88 ± 0.39 ^A^	2.86 ± 1.06 ^B^	3.49 ± 0.43 ^AB^	3.7 ± 0.91 ^A^	3.65 ± 0.53 ^A^
Ethyl E-11-hexadecenoate	n.f.	0.2 ± 0.14 ^B^	0.19 ± 0.08 ^B^	0.15 ± 0.01 ^B^	0.15 ± 0.05 ^B^	0.36 ± 0.04 ^B^	0.72 ± 0.06 ^A^
Ethyl 9-decenoate	Fruity, fatty	0.34 ± 0.07 ^B^	0.36 ± 0.06 ^B^	0.5 ± 0.02 ^B^	0.43 ± 0.08 ^B^	0.95 ± 0.18 ^A^	1.25 ± 0.29 ^A^
Hexanoic acid ethyl ester	n.f.	0.87 ± 0.42 ^C^	1.57 ± 0.14 A^B^	1.12 ± 0.53 ^BC^	1.78 ± 0.71 ^A^	1.67 ± 0.38 ^A^	1.42 ± 0.33 ^AB^
2-methylbutyl valerate	n.f.	2.46 ± 0.64 ^B^	3.76 ± 0.88 ^A^	3.12 ± 0.74 ^A^	3.86 ± 0.92 ^A^	2.31 ± 0.31 ^B^	2.09 ± 0.38 ^B^
Methyl 2-methylhexanoate	n.f.	0.52 ± 0.03 ^A^	n.d.	0.18 ± 0.07 ^B^	n.d.	0.04 ± 0.01 ^B^	0.14 ± 0.01 ^B^
Ethyl octadecanoate	Waxy	n.d.	n.d.	0.04 ± 0.01 ^A^	n.d.	0.02 ± 0.01 ^A^	0.06 ± 0.02 ^A^
Ethyl octanoate	Fruity, wine, waxy, sweet, apricot, banana, brandy, pear	2.15 ± 0.14 ^E^	2.97 ± 0.84 ^D^	4.34 ± 1.03 ^C^	4.56 ± 1.33 ^C^	7.96 ± 3.01 ^A^	7.13 ± 0.95 ^B^
Pentanoic acid, 2,2-dimethyl-methyl ester	n.f.	2.52 ± 0.66 ^AB^	2.88 ± 0.13 ^A^	1.66 ± 0.71 ^C^	1.89 ± 0.54 ^BC^	1.75 ± 0.21 ^BC^	1.83 ± 0.73 ^B^
Pentanoic acid, 2,4-dimethyl-methyl ester	n.f.	n.d.	0.31 ± 0.03 ^A^	n.d.	0.14 ± 0.09 ^B^	n.d.	0.19 ± 0.05 ^B^
Pentanoic acid, 2-methyl-butyl ester	n.f.	0.03 ± 0.02 ^A^	0.04 ± 0.01 ^A^	0.05 ± 0.02 ^A^	0.02 ± 0.01 ^A^	0.09 ± 0.01 ^A^	0.02 ± 0.01 ^A^
Methyl vanillate	Warm, spicy, vanilla, guaiacol, phenolic, carnation	n.d.	n.d.	0.65 ± 0.01 ^D^	1.12 ± 0.34 ^C^	1.55 ± 0.51 ^B^	1.74 ± 0.35 ^A^
Ethyl vanillate	Phenolic, burnt, guaiacol, smoky, powdery, metallic	n.d.	n.d.	2.65 ± 0.13 ^C^	3.12 ± 1.04 ^B^	3.78 ± 1.27 ^A^	3.81 ± 1.38 ^A^
Diethyl succinate	Mild, fruity, cooked apple, ylang	0.47 ± 0.11 ^D^	0.66 ± 0.24 ^D^	2.34 ± 0.17 ^C^	3.12 ± 1.19 ^B^	3.55 ± 1.93 ^A^	3.14 ± 1.16 ^B^
**TOTAL**		**17.79**	**22.93**	**24.97**	**29.63**	**34.8**	**36.45**
	**Thiols**						
1-Propene-1-thiol	n.f.	0.41 ± 0.12 ^A^	0.32 ± 0.09 ^A^	0.29 ± 0.04 ^A^	0.31 ± 0.07 ^A^	0.33 ± 0.01 ^A^	0.33 ± 00.09 ^A^
**TOTAL**		**0.41**	**0.32**	**0.29**	**0.31**	**0.33**	**0.31**
	**Ketones**						
3,3-Dimethyl-4-methylamino-butan-2-one	n.f.	0.04 ± 0.01 ^A^	0.05 ± 0.01 ^A^	0.06 ± 0.04 ^A^	0.05 ± 0.03 ^A^	0.04 ± 0.02 ^A^	0.05 ± 0.13 ^A^
Β-damascenone	Sweet, fruity, rose, plum, grape, raspberry, sugar	0.0005 ± 0.0001 ^A^	0.0004 ± 0.0002 ^A^	0.0005 ± 0.0002 ^A^	0.0005 ± 0.0003 ^A^	0.0005 ± 0.0001 ^A^	0.0006 ± 0.0002 ^A^
**TOTAL**		**0.0405**	**0.0504**	**0.0605**	**0.0505**	**0.0405**	**0.0506**
	**Terpenes**						
Linalool	Citrus, floral, sweet, bois de rose, woody, green, blueberry	0.45 ± 0.15 ^C^	0.98 ± 0.39 ^BC^	1.77 ± 0.48 ^AB^	1.98 ± 0.62 ^A^	2.12 ± 1.04 ^A^	2.32 ± 0.94 ^A^
Citronellol	Floral, leather, waxy, rose, bud, citrus	1.23 ± 0.04 ^B^	1.12 ± 0.61 ^B^	1.98 ± 0.83 ^A^	1.73 ± 0.55 ^AB^	1.84 ± 0.73 ^AB^	1.91 ± 0.58 ^A^
Geraniol	n.f.	0.44 ± 0.11 ^B^	0.69 ± 0.17 ^A^	0.12 ± 0.04 ^B^	0.22 ± 0.09 ^B^	0.18 ± 0.08 ^B^	0.16 ± 0.03 ^B^
Eugenol	Sweet, spicy, clove, woody	1.16 ± 0.01 ^A^	1.12 ± 0.03 ^A^	0.43 ± 0.06 ^B^	0.65 ± 0.12 ^B^	0.98 ± 0.31 ^AB^	0.77 ± 0.11 ^AB^
**TOTAL**		**3.28**	**3.91**	**4.3**	**4.58**	**5.12**	**5.16**
	**Phenols**						
4-vinylguaiacol	Sweet, spicy, clove-like, somewhat smoky	n.d.	n.d.	0.65 ± 0.13 ^AB^	0.32 ± 0.07 ^B^	0.98 ± 0.24 ^A^	0.44 ± 0.14 ^B^
**TOTAL**		**n.d.**	**n.d.**	**0.65**	**0.32**	**0.98**	**0.44**
		**Aldehydes**					
Furfural	Almond, wood, caramel	n.d.	n.d.	0.87 ± 0.02 ^D^	0.98 ± 0.03 ^C^	1.28 ± 0.02 ^B^	1.43 ± 0.02 ^A^
Vanillin	Sweet, vanilla, creamy, chocolate	n.d.	n.d.	1.56 ± 0.23 ^B^	2.74 ± 0.03 ^B^	3.78 ± 0.76 ^A^	3.98 ± 0.04 ^A^
**TOTAL**		**n.d.**	**n.d.**	**2.46**	**3.72**	**5.06**	**5.41**
		**Lactones**					
Butyrolactone	Milky, creamy, fruity, peach	n.d.	n.d.	0.91 ± 0.36 ^A^	0.98 ± 0.45 ^A^	1.12 ± 0.34 ^A^	0.87 ± 0.27 ^A^
Trans-whiskey lactone	n.f.	n.d.	n.d.	1.88 ± 0.47 ^A^	1.66 ± 0.63 ^A^	1.98 ± 0.77 ^A^	1.87 ± 0.55 ^A^
**TOTAL**		**n.d.**	**n.d.**	**2.79**	**2.64**	**3.1**	**2.74**

n.f.: not found; n.d.: not determined; different letters in the same line indicate significant differences (*p* < 0.05).

## 4. Conclusions

The careful selection of yeast strains, combined with an appropriate inoculation strategy, is an essential requirement to guarantee an accurate fermentation process and to obtain novel wine style. On the basis of the obtained data, the exploitation of oak chips and oak surface-adhered *Starm. bacillaris* cells seems to be a good approach to improve the volatile profile of Trebbiano d’Abruzzo wines and their sensory traits. The use of oak chips to modulate the characteristics of white wine is relatively new. Further studies are necessary to evaluate the effect of non-oak wood species for the production of Trebbiano d’Abruzzo wines in order to produce wines with potential new oenological characteristics and to valorize this cultivar.

## Figures and Tables

**Table 1:** Fermentation trials performed in this study.

Trial	Strains	Type of Inoculum
W1	RT73 + CZ31	CO
W2	RT73 + CZ31	SQ
W3	RT73 + CZ31	CO+ oak chips
W4	RT73 + CZ31	SQ+ oak chips
W5	RT73 + CZ31	CO+ CZ31 adhered to oak chips
W6	RT73 + CZ31	SQ+ CZ31 adhered to oak chips

**Table 2:** Main oenological parameters of obtained wines.

Trial	Alcohol (% *v*/*v*)	Residual Sugars (g/L)	pH	Total Acidity (g/L) *	Volatile Acidity (g/L) **	Glycerol (g/L)	Polyphenols (g/L)
W1	11.07 ± 1.23 ^A^	0.51 ± 0.11 ^A^	3.48 ± 0.45 ^A^	6.05 ± 1.09 ^A^	0.56 ± 0.08 ^A^	5.19 ± 2.43 ^B^	126 ± 37.89 ^C^
W2	11.07 ± 2.89 ^A^	0.58 ± 0.12 ^A^	3.52 ± 0.77 ^A^	6.04 ± 1.22 ^A^	0.56 ± 0.14 ^A^	5.18 ± 1.88 ^B^	183 ± 58.43 ^C^
W3	11.04 ± 0.98 ^A^	0.59 ± 0.09 ^A^	3.48 ± 0.26 ^A^	6.12 ± 2.09 ^A^	0.54 ± 0.06 ^A^	5.33 ± 1.05 ^B^	223 ± 88.43 ^B^
W4	11.13 ± 1.55 ^A^	0.61 ± 0.17 ^A^	3.56 ± 0.84 ^A^	6.11 ± 0.87 ^A^	0.52 ± 0.04 ^A^	5.15 ± 0.62 ^B^	220 ± 91.84 ^B^
W5	11.06 ± 2.06 ^A^	0.59 ± 0.06 ^A^	3.51 ± 0.98 ^A^	5.99 ± 0.44 ^A^	0.57 ± 0.18 ^A^	6.53 ± 1.29 ^A^	330 ± 73.54 ^A^
W6	11.07 ± 1.22 ^A^	0.58 ± 0.04 ^A^	3.54 ± 0.37 ^A^	6.02 ± 0.35 ^A^	0.55 ± 0.09 ^A^	6.85 ± 1.84 ^A^	344 ± 81.45 ^A^

Different letters in the same column indicate significant differences (*p* < 0.05); * expressed as tartaric acid; ** expressed as acetic acid.

**Table 3:** Effect of *S. cerevisiae* RT73 and *Starm. bacillaris* CZ31 strains and oak chips on the chromatic characteristics of Trebbiano d’Abruzzo wines.

Trial	L*	a*	b*	C*	H*	Intensity
W1	97.44 ± 1.34 ^B^	−0.25 ± 0.04 ^B^	2.07 ± 0.87 ^C^	2.07 ± 0.54 ^C^	95.22 ± 1.98 ^C^	0.11 ± 0.12 ^B^
W2	97.41 ± 6.87 ^B^	−0.21 ± 0.06 ^B^	2.25 ± 0.91 ^C^	2.26 ± 0.36 ^C^	96.37 ± 1.49 ^C^	0.12 ± 0.22 ^B^
W3	97.99 ± 3.66 ^A^	−0.27 ± 0.03 ^B^	2.56 ± 0.5 ^B^	2.57 ± 0.55 ^B^	97.05 ± 1.45 ^B^	0.13 ± 0.08 ^B^
W4	98.28 ± 5.12 ^A^	−0.31 ± 0.05 ^B^	2.68 ± 0.77 ^B^	2.71 ± 0.13 ^B^	97.66 ± 1.87 ^B^	0.19 ± 0.45 ^A^
W5	98.21 ± 2.77 ^A^	−0.41 ± 0.02 ^A^	3.04 ± 0.27 ^A^	3.07 ± 0.54 ^A^	98.59 ± 0.99 ^A^	0.15 ± 0.17 ^B^
W6	98.25 ± 7.98 ^A^	−0.39 ± 0.06 ^A^	3.45 ± 0.62 ^A^	3.07 ± 0.98 ^A^	98.31 ± 1.32 ^A^	0.18 ± 0.15 ^A^

Different letters in the same column indicate significant differences (*p* < 0.05).

## Data Availability

The datasets generated during and/or analyzed during the current study are available from the corresponding author on reasonable request.
